# Editorial: RNA-Mediated Epigenetic and Transcriptional Regulation

**DOI:** 10.3389/fgene.2022.928335

**Published:** 2022-06-06

**Authors:** A. Rasim Barutcu, Yicheng Long, Mo Motamedi

**Affiliations:** ^1^ Donnelly Centre for Cellular and Biomolecular Research, University of Toronto, Toronto, ON, Canada; ^2^ Department of Biochemistry, Cardiovascular Research Institute, Weill Cornell Medicine, New York, NY, United States; ^3^ Massachusetts General Hospital Center for Cancer Research and Department of Medicine, Harvard Medical School, Charlestown, MA, United States

**Keywords:** RNA, transcription, RNA modification, miRNA, RNA epigenetic regulation

RNA plays major regulatory roles in many processes beyond transcription, including protein complex formation, maintenance of genomic stability, and establishment of higher-order cellular structures *via* phase separation. Accordingly, regulation of RNA abundance, processing, and modification can have a profound impact on cellular and organismal biology. In recent years, significant progress has been made to decipher the complex molecular details and functional interplay between RNA and regulatory processes have begun to emerge. These studies have shed light on how perturbation to these mechanisms can yield aberrant biology, including formation of disease states such as cancers. In this Research Topic “*RNA-Mediated Epigenetic and Transcriptional Regulation*,” several articles highlight the importance of RNA-mediated regulation in different pathological contexts such as cancers and retinal degeneration, and developmental/adaptation processes such as sperm development, resistance to pathogens, and adaptation to cold. Altogether, this Research Topic further highlights the importance of RNA as an effector molecule in biology and underscores its biochemical versatility as a regulatory molecule.

N^6^-methyladenosine (m6A) is the most prevalent, conserved and abundant co-transcriptional modification observed in eukaryotic mRNAs. It plays important roles in splicing, translation, RNA stability, and higher-order RNA structures (Jiang et al., 2021) among other biological processes. There are several regulatory complexes which “write,” “erase,” and “read” this modification on RNA molecules. The delicate regulatory control of m6A has been implicated in numerous physiological and pathological conditions, especially in cancers. In this Research Topic, three research papers and a review paper evaluate, identify, and summarize the m6A landscape of several cancers, including melanoma, acute myeloid leukemia (AML), adenocarcinoma of the esophagogastric junction (AEG) and bladder cancer. Du et al. performed computational analyses on over one thousand melanoma patient samples to determine whether there is a relationship between m6A modification and melanoma immunogenicity. By performing clustering analyses and devising a specialized m6A enrichment scoring system, the authors identified distinct expression profiles of m6A machinery and m6A modification patterns that correlate with known immune phenotypes in melanoma. Li et al. used The Cancer Genome Atlas (TCGA) AML cohort and identified seven long non-coding RNAs (lncRNAs), whose expression not only correlated with at least one m6A regulator, but also could be used as prognostic markers for AML and its immunotherapy response in patients. By using matched tumor and normal tissues Huang et al., performed m6A sequencing to generate an m6A map of AEG. Their work identified several AEG-specific m6A sites on important cancer-related mRNAs, suggesting their importance in this cancer. Finally, Liu reviewed m6A modification and its potential regulatory role in bladder cancer proliferation and infiltration. Altogether, these articles showcase m6A dynamics and its potential impact on a disease state such as cancer.

Recent studies have shown that m6A modification also plays important roles in organismal development. Dang et al., assessed the differences in the transcriptomes and m6A profiles of two distinct cattle breeds, differentiated based on their muscle composition. They identified several differentially m6A-modified mRNAs important in muscle development and related pathways. These data suggest that differential m6A modification of these mRNAs could play a role in the distinct muscle profiles found in these cattle breeds. In another report, Liu et al. investigated the role of m6A modification in bovine spermatogenesis during three developmental stages (pre-puberty, puberty, and post-puberty). Their analyses revealed important genes and developmental pathways display differential RNA expression and m6A profiles at distinct ages, correlating m6A regulation with sperm development.

RNA regulation also has a profound impact on development and tuning the metabolic state of organisms, especially in response to stress. Focusing on retinal development, Wei et al. performed RNA-seq on tissues obtained from three different mouse models for inherited retinal degeneration (RD) and identified important regulatory networks such transcription factors, lncRNAs and circular RNAs, whose expression change in RD. In another study Zheng et al., performed transcriptomic and metabolomic profiling of a yak breed during the cold season and generated an integrative transcriptome and metabolome map revealing several differentially regulated metabolic pathways which could help the animals survive long periods of malnutrition and cold temperatures.

MicroRNAs (miRNAs) constitute a highly conserved family of eukaryotic regulatory small non-coding RNAs, typically 19–24 nucleotides in length, formed by cleavage of endogenous hairpin non-coding RNAs. They mediate silencing of the complementary transcripts by promoting degradation or inhibiting translation of the complementary transcripts. They function in a myriad of developmental and pathological processes. Further implicating the importance of RNA-mediated gene regulation, Yang et al. investigated the role of miRNAs in pesticide resistance of the diamondback moth, which is one of the deadliest and most destructive pests worldwide. By performing small RNA sequencing from the guts of drug-resistant and sensitive moths, the authors identify dozens of differentially expressed miRNAs whose predicted target genes may be involved in pesticide resistance.

Altogether, these descriptive studies further establish the link between RNA and its regulatory potential in governing a wide array of biological processes. They also underscore the importance of answering several outstanding mechanistic questions about how cells harness the unique biochemical properties of RNA to mediate mechanisms impacting many important biological activities. Indeed, such molecular insight will not only extend our understanding of the basic biology of cellular processes, but also help us develop potential therapeutics for several diseases.
